# Prostate specific antigen (PSA) kinetic as a prognostic factor in metastatic prostate cancer receiving androgen deprivation therapy: systematic review and meta-analysis

**DOI:** 10.12688/f1000research.14026.1

**Published:** 2018-02-28

**Authors:** Andika Afriansyah, Agus Rizal Ardy Hariandy Hamid, Chaidir Arif Mochtar, Rainy Umbas

**Affiliations:** 1 Department of Urology, Cipto Mangunkusumo Hospital, Faculty of Medicine, University of Indonesia, Jakarta, 10430, Indonesia

**Keywords:** androgen deprivation therapy, metastasis, PSA kinetics, prostate cancer, survival, systematic review, meta analysis

## Abstract

**Aim: **Metastatic prostate cancer (mPCa) has a poor outcome with median survival of two to five years. The use of androgen deprivation therapy (ADT) is a gold standard in management of this stage.  Aim of this study is to analyze the prognostic value of PSA kinetics of patient treated with hormonal therapy related to survival from several published studies

**Method: **Systematic review and meta-analysis was performed using literature searching in the electronic databases of MEDLINE, Science Direct, and Cochrane Library. Inclusion criteria were mPCa receiving ADT, a study analyzing Progression Free Survival (PFS), Overall Survival (OS), or Cancer Specific Survival (CSS) and prognostic factor of survival related to PSA kinetics (initial PSA, PSA nadir, and time to achieve nadir (TTN)). The exclusion criteria were metastatic castration resistant of prostate cancer (mCRPC) and non-metastatic disease. Generic inverse variance method was used to combine hazard ratio (HR) within the studies. Meta-analysis was performed using Review Manager 5.2 and a p-value <0.05 was considered statistically significant.

**Results: **We found 873 citations throughout database searching with 17 studies were consistent with inclusion criteria. However, just 10 studies were analyzed in the quantitative analysis. Most of the studies had a good methodological quality based on Ottawa Scale. No significant association between initial PSA and PFS. In addition, there was no association between initial PSA and CSS/ OS. We found association of reduced PFS (HR 2.22; 95% CI 1.82 to 2.70) and OS/ CSS (HR 3.31; 95% CI 2.01-5.43) of patient with high PSA nadir. Shorter TTN was correlated with poor result of survival either PFS (HR 2.41; 95% CI 1.19 – 4.86) or CSS/ OS (HR 1.80; 95%CI  1.42 – 2.30)

**Conclusion: **Initial PSA before starting ADT do not associated with survival in mPCa.  There is association of PSA nadir and TTN with survival

## Introduction

Prostate cancer (PCa) is the second most common cancer in men, and the fourth most common cancer worldwide. More than one million men worldwide were diagnosed with PCa in 2012 ^1^. The incidence of local-regional PCa has increased since the introduction of prostate specific antigen (PSA). This circumstance reduces the incidence of metastatic PCa ^2^. PCa patient treated at early stages have a good prognosis with 5-year overall survival (OS) reaching 99%. In contrast, metastatic PCa patients generally experience a poor outcome. Several published studies showed a wide difference of survival, with median OS from two to five years ^3– 5^. Androgen deprivation therapy (ADT) becomes the standard treatment of patients with advanced PCa ^6, 7^, and with the first use reported by Huggins and Hodges in 1941 ^8^.

In clinical practice, PSA is the most common diagnostic procedure to evaluate the disease and to predict the survival. PSA kinetics such as nadir PSA level, time to reach nadir (TTN), or specific PSA value after initiation of ADT might became a predictor of survival in several retrospective and clinical trial studies ^5, 9– 11^. Some limitations were shown in the previous report of investigation for PSA kinetic to survival. They included patients with heterogeneous backgrounds (such as metastatic disease prior to surgical or radiation therapy), and the sample size was small. Therefore, we performed a systematic review and meta-analysis to evaluate the pooled effect of PSA kinetics of patient treated with hormonal therapy related to survival from several published studies.

## Methods

### Eligibility criteria

The systematic review was performed according to Preferred Reporting Items for Systematic Reviews and Meta-Analyses (PRISMA) guidelines ^12^. All studies in English were included. Retrospective cohorts, prospective cohort, randomized clinical trial (RCT), were eligible for inclusion for this review. The inclusion criteria were that (i) the participant of the study had metastatic PCa; (ii) patients were treated with ADT either using orchiectomy or luteinizing hormone-releasing hormone (LHRH) agonist with or without anti-androgen (AA); (iii) the studies outcome were either progresion free survival (PFS), overall survival (OS) or cancer specific survival (CSS); (iv) the studies had to analyze PSA kinetics (intial PSA prior to initiation of ADT, PSA nadir, and time to reach nadir (TTN) PSA). Studies analyzed in meta-analysis had to use adjusted analysis of prognostic factors, such as multivariate Cox regression, to overcome the confounding factors. Studies that analyzed patient with castration resistant PCa (CRPC) and non-metastatic disease were excluded.

### Search strategy

Electronic searched were performed in three databases: MEDLINE, Science Direct, and Cochrane Library from 1950 to 2016. This literature searching was conducted in March 2017. Gray literature and conference abstract, especially from urology oncology conference, were also searched. References list from included article were reviewed. We used the following search strategy: (prostate cancer OR adenocarcinoma prostate), (survival OR prognosis OR prognostic), (metastasis OR metastases OR metastatic), (PSA OR “Prostate Specific Antigen” OR nadir OR “initial PSA” OR kinetic). Two researchers (A.A and A.R.A.H) were indecently assessing the title and abstract of the paper. They agreed the studies included in the meta-analysis. Disagreement between the two review authors on the selection of studies was resolved by discussion with third authors (C.A.M) as a senior investigator. We used EndNote X6 for screening of duplicated studies.

### Data extraction and quality assessment

A data extraction table was created to extract data from each article. The data of study design, patient's characteristics, method of ADT, duration of follow up, outcomes of survival, and significant prognostic factors of PSA kinetics were collected from all included studies. For the observational studies, the quality of study was assessed using Newcastle-Ottawa Scale (NOS). There were three major components of this scale namely the selection of the group of the study, comparability, and assessment of the outcome. The quality of study assessed with number of stars based on NOS. A maximum 7 stars could be scored; 6 or 7 stars considered as high quality study, 4 – 5 stars corresponded with intermediate quality, and 0 – 3 stars showed low quality ^13^.

### Synthesis of results

Meta-analysis was applied on studies with prognostic factor with similar outcome definition. I ^2^ test was conducted in order to evaluate the heterogeneity, whilst for >30% a random effects model was applied, or otherwise, fixed effects model was done. Confounding in the individual studies was estimated using Hazard Ratio (HR) adjusted estimation, thus generic inverse variance method was used. We only combined data to estimate pooled effect of categorical parameters due to feasibility of statistical analysis. Studies that evaluated parameters but could not synthesize to meta-analysis were describe quantitatively. Meta-analysis was performed using Review Manager 5.2 from Cochrane Collaboration. A p-value < 0.05 was considered statistically significant.

## Results

**Figure 1.**
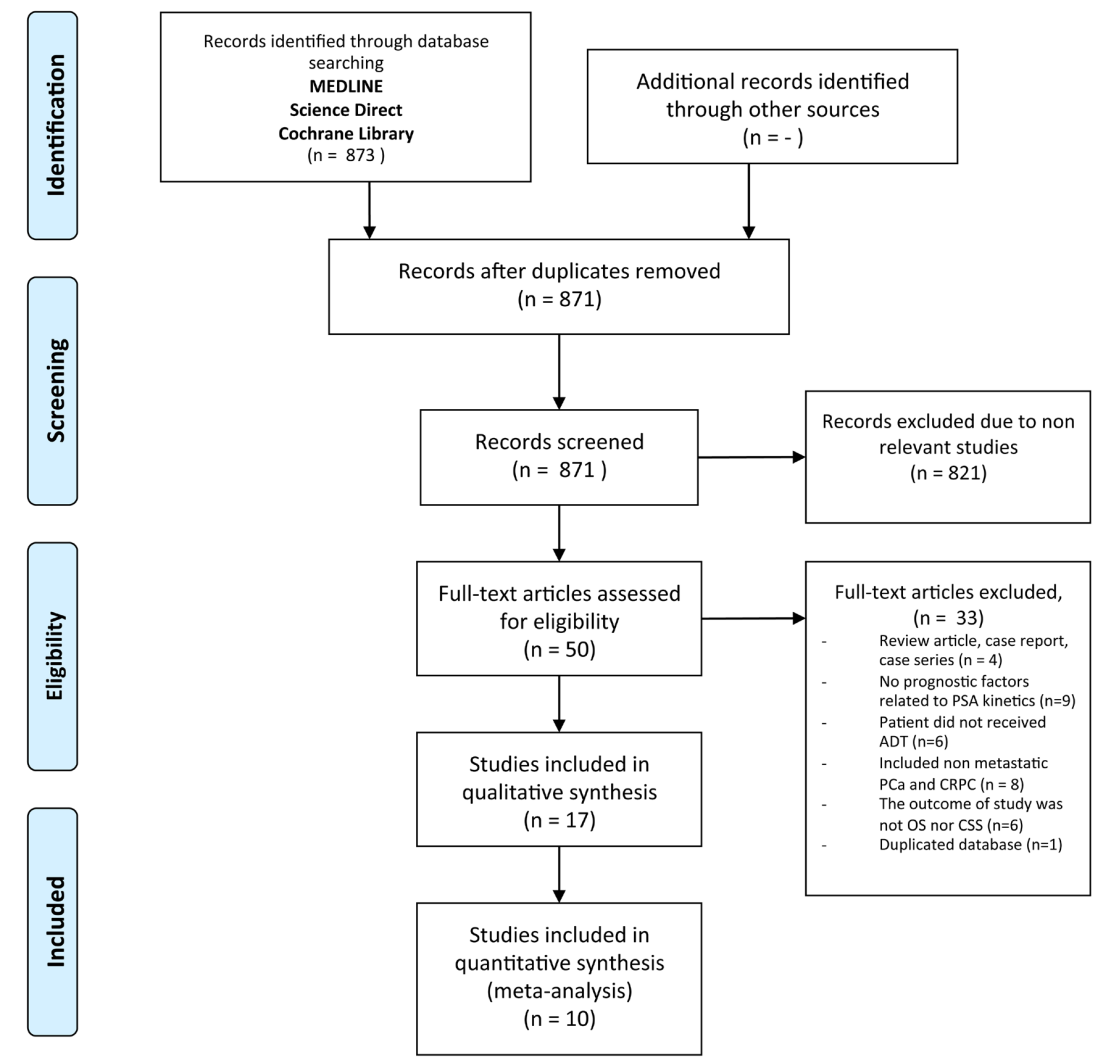
Flowchart showing the searching strategy of the studies.

We found 873 citations throughout database searching. No additional records identified through searching from reference list of included studies. Seventeen studies were found to be consistent to the inclusion criteria of the study, but seven studies could not be evaluated the in meta-analysis ( Figure 1). Miyamoto *et al.* ^14^ did not published the hazard ratio, and put the cumulative survival rate as the outcome. Six other studies used numerical parameters of PSA kinetics that cannot combine in the forest plot ^9, 15– 19^. All of those studies were considered in qualitative synthesis. The characteristic of study is present in Table 1. Based on NOS, the quality of study included was good ( Table 2).

**Table 1.** Characteristic of included studies.

Study	Total patient	Androgen Deprivation Therapy	Follow - up time	Survival outcome	Significance Prognostic Factor
Bello 2017 ^25^	M1 = 79	• Orchiectomy • LHRH agonist	NM	Median OS was 40.3 months	• NSAID use • PSA nadir
Choueiri 2009 ^5^	M1 = 179	• LHRH agonist with or without AA • Orchiectomy	Median follow up 48 months	Median OS was 84 months	• GS • TTN • PSA Nadir
Glass 2003 ^9^	M1 = 1,076	Bilateral orchiectomy with or without AA (flutamide)	NM	Median OS was 32 months	• Initial PSA • Presence appendicular bone disease • GS • Presence of bone pain • PS
Hong 2012 ^10^	M1 = 131	CAB using LHRH agonist plus AA	Median follow up 30 months	Median CSS • PSA nadir < 2 ng/ml was 91.7 months • PSA nadir ≥ 2 ng/ml 49.8	• PSA nadir • TTN
Hussain 2006 ^11^	M1 = 1,345	CAB using LHRH agonist (gosereline) plus AA (bicalutamide)	Median follow up was 38.0 months	Median OS of PSA after 7 months ADT • ≤ 0.2 ng/ml was 75 months • 0.2 < PSA ≤ 0.2 ng/ml was 44 months • PSA > 4.0 ng/ml was 13 months	• PSA after 7 months of ADT • ECOG • Presence of bone pain • GS
Kadono Y, 2015 ^4^	M1a = 224, M1b = 4386, M1c =278	• LHRH agonist with or without AA • Orchiectomy	Mean follow up 3.3 years	The 5-year OS was • 57.5% in M1a • 54.0% in M1b • 40.0% in M1c	• GS • PSA • Age
Kim KH, 2015 ^18^	M1 = 398	• CAB (LHRH agonist plus AA)	Median follow up 44 months	Median CSS was 65 months	• GS • PSA nadir • TTN • PSA Half Life • N1
Kimura, 2014 ^24^	M1 = 3006	• Type of ADT was not clear	Median followed up in young, middle and elderly group was 25.5, 35.3 and 38.5 months	The 5-years OS • Young age was 26.6% • Middle age was 59.7% • Elderly age was 55.3%	• GS • Concomitant bone and visceral metastasis • Age • Clinical T
Koo, 2014 ^17^	M1b = 248	• Type of ADT was not clear	Median follow up 39.3 months	Median CSS in PSA nadir • < 0.2 ng/ml was 70 months • ≥ 0.2 ng/ml was 50 months	• PSA nadir • ALP • ECOG
Kwak 2002 ^23^	M1 = 145	• LHRH agonist with or without AA • Orchiectomy	Median follow up 39 months	Median survival of patients with Nadir PSA (months) • < 0.2, = 53 • 0.2 to 1.0 = 42 • 1.1 to 10 = 24 • >10.1 = 15	• Nadir PSA
Miller 1992 ^25^	M1 = 48 patients	• Orchiectomy • LHRH agonist • Diethylatibestrol	Median follow up 42 months	Median PFS 19 months	• Nadir PSA
Miyamoto ^14^	M1 – 94	• LHRH agonist with AA	Median follow up 38.8 months	5-yr OS rate 62.5%	• PSA • Gleason Grade
Nayyar 2010 ^16^	M1 = 412	• Surgical castration • Medical castration • Antiandrogen	Median follow up 55 months	Median OS 5.7 years	• GS • PSA doubling time
Park, 2009 ^15^	M1 = 131	• LHRH agonist with or without AA • Orchiectomy	Median follow up was 53.0 months	Median CSS • Short PSA doubling time was 35 months • Long PSA doubling time 95 months	• High Nadir PSA • Short PSA half time • Short PSA doubling time after nadir
Sasaki 2011 ^22^	M1 = 412	• Bilateral orchiectomy • LHRH agonist	NM	Median OS 5.7 years	• PSA half time • PSA doubling time • GS
Teoh 2017 ^21^	M1b = 419	• LHRH agonist • Bilateral orchiectomy	Median follow up was 48 months	Median OS was 28 months	• PSA nadir ≥ 2 ng/ml • TTN < 9.09 months
Tomiokoa 2014 ^20^	M1 = 236	• LHRH agonist • surgical castration • AA monotherapy • CAB	Median follow up 47 months	The 5-years OS was 63%	• Nadir PSA ≥ 0.2 ng/ml • TTN < 6 month

ADT = androgen deprivation therapy; LHRH = luteinizing hormone releasing hormone; AA = anti androgen; CAB = combined anti androgen; PSA = prostate specific antigen; OS = overall survival; GS = Gleason score; TTN = time to nadir; NM = not mentioned; NSAID = non-steroidal anti inflammatory drug; ECOG PS = Eastern Cooperative Oncology Group Performance Status; PS = Performance Status; ALP = alkaline phosphatase; N1= regional nodal metastasis

**Table 2.** Methodological quality of the study based on NOS Scale.

Study	Selection (Max ****)				Comparability (Max **)	Outcome (Max ***)			Total Score
	Representativeness of exposed cohort	Selection of exposed cohort	Ascertainment of exposure	No outcome of interest at start	Comparability of cohorts on the basis of design of analysis	Assessment of outcome	Was follow-up long enough for outcomes to occur	Adequacy of follow up of cohorts	
Bello 2017 ^25^	*	*	*	*	**	*	Not clear	Not clear	7
Choueiri 2009 ^5^	*	*	*	*	*	*	*	*	9
Glass 2003 ^9^	*	*	*	*	**	*	Not clear	Not clear	7
Hong 2012 ^10^	*	*	*	*	**	*	*	*	9
Hussain 2006 ^11^	*	*	*	*	**	*	*	*	9
Kadono 2015 ^4^	*	*	*	*	**	*	*	*	9
Kim 2015 ^18^	*	*	*	*	**	*	*	*	9
Kimura 2014 ^24^	*	*	*	*	*	*	*	*	8
Koo 2014 ^17^	*	*	*	*	**	*	*	*	9
Kwak 2002	*	*	*	*	**	*	*	*	9
Miller 1992 ^19^	*	*	*	*	*	*	*	*	8
Miyamoto 2012 ^14^	*	*	*	*	**	*	*	*	9
Nayyar 2010	*	*	*	*	**	*	*	*	9
Park 2009 ^15^	*	*	*	*	**	*	*	*	9
Sasaki 2011 ^22^	*	*	*	*	**	*	-	*	7
Teoh 2017 ^21^	*	*	*	*	**	*	*	*	9
Tomioka 2014 ^20^	*	*	*	*	**	*	*	*	9

### Evaluation of PSA kinetics

**Figure 2.**
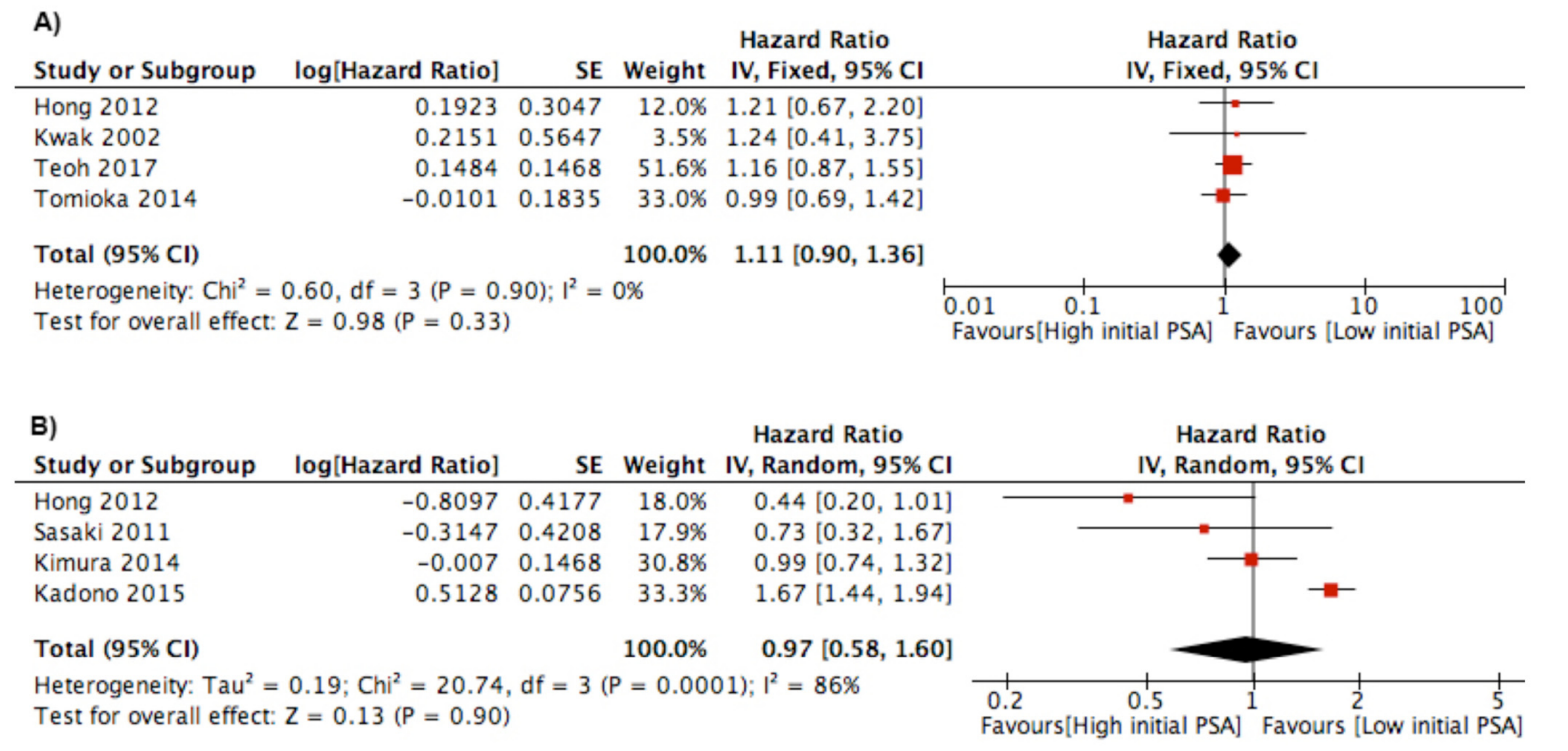
Forest plot of association between initial PSA and: **A**) Progression Free Survival Outcome; **B**) Cancer Specific Survival/Overall Survival.

***Initial PSA***. Initial PSA before ADT treatment was evaluated in twelve studies ^4, 9– 11, 16, 17, 19– 25^. However, we only put four studies in PFS outcome and four studies in CSS/OS outcome because the studies analyzed initial PSA as a categorical parameter. No significant association between initial PSA and PFS was found, and the studies were homogenous (I ^2^=0%). In addition, there was no association between initial PSA and CSS/OS ( Figure 2). In qualitative analysis, four studies analyzed the association between initial PSA and PFS. All of the studies did not find significant results for PSA and PFS ^16, 17, 19, 25^. The result was the same when we analyzed the studies for OS/CSS outcome ^9, 11, 16^.

**Figure 3.**
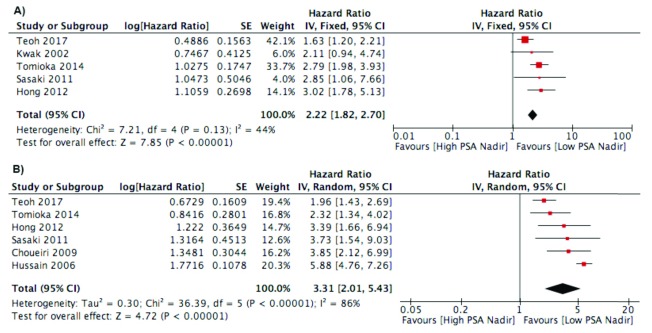
Forest plot of association between PSA nadir and: **A**) Progression Free Survival Outcome; **B**) Cancer Specific Survival/Overall Survival.

***PSA Nadir***. Six studies analyzed the effect of PSA nadir to influence survival using 0.2 ng/ml as a cut-off point ^5, 10, 11, 20, 22, 23^. Four studies analyzed the PSA nadir as a continuous variable ^15, 17– 19^. Teoh *et al.* used cut-off point 2 ng/ml as a PSA nadir that influence the survival ^21^. Bello *et al.* analysed nadir using 4 ng/ml as a threshold ^25^. Meta-analysis of the studies found an association of reduced PFS of patient with high PSA nadir (HR 2.22; 95% CI 1.82 to 2.70). The studies appear homogenous in the forest plot. In addition, high PSA nadir had a negative impact on the OS/CSS outcome with HR 3.31 (95% CI 2.01–5.43) ( Figure 3). In the studies using continuous measurement of PSA nadir, three studies found significant association of nadir PSA and survival ^15, 18, 19^. However, studies by Koo *et al.* found no significant result ^17^. Miyamoto *et al.* found the PSA nadir after first line hormonal therapy influenced survival ^14^.

**Figure 4.**
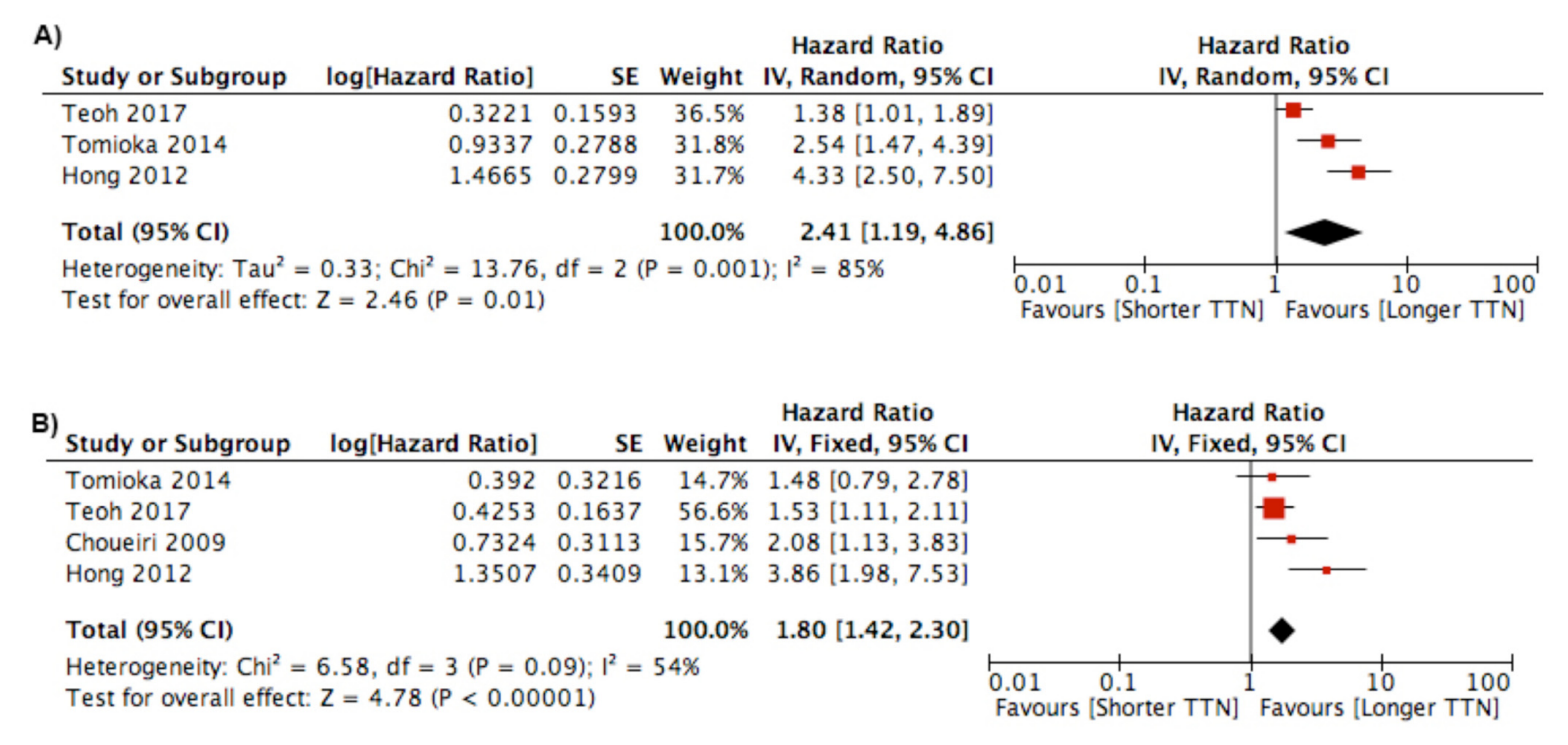
Forest plot of association between TTN and: **A**) Progression Free Survival Outcome; **B**) Cancer Specific Survival/Overall Survival.

***Time to Nadir (TTN)***. A total of seven studies analyzed the relationship between TTN and survival ^5, 10, 16– 18, 20, 21^. Of the seven studies, two studies used 8 months ^5, 10^, one study used 9 months ^26^, and one study used 12 months ^20^ as a cut-off. Three studies analyzed TTN as a continuous variable ^16– 18^. Meta-analysis was performed with showing a shorter TTN correlated with poor survival for both PFS (HR 2.41; 95% CI 1.19 – 4.86) or CSS/OS (HR 1.80; 95%CI 1.42 – 2.30) ( Figure 4). Studies using continuous variable of TTN showed a significant negative effect from shorter TTN on survival.

Dataset 1. Quality assessment (based on NOS), hazard ratio, and standard error of studies included in initial PSA parameter http://dx.doi.org/10.5256/f1000research.14026.d195553
For quality assessment a maximum 7 stars could be scored; 6 or 7 stars considered as high quality study, 4 – 5 stars corresponded with intermediate quality, and 0 – 3 stars showed low quality. Click here for additional data file. Copyright: © 2018 Afriansyah A et al. 2018 Data associated with the article are available under the terms of the Creative Commons Zero "No rights reserved" data waiver (CC0 1.0 Public domain dedication).

Dataset 2. Quality assessment (based on NOS), hazard ratio, and standard error of studies included in PSA nadir parameter http://dx.doi.org/10.5256/f1000research.14026.d195573
For quality assessment a maximum 7 stars could be scored; 6 or 7 stars considered as high quality study, 4 – 5 stars corresponded with intermediate quality, and 0 – 3 stars showed low quality. Click here for additional data file. Copyright: © 2018 Afriansyah A et al. 2018 Data associated with the article are available under the terms of the Creative Commons Zero "No rights reserved" data waiver (CC0 1.0 Public domain dedication).

Dataset 3. Quality assessment (based on NOS), hazard ratio, and standard error of studies included in time to nadir parameter http://dx.doi.org/10.5256/f1000research.14026.d195577
For quality assessment a maximum 7 stars could be scored; 6 or 7 stars considered as high quality study, 4 – 5 stars corresponded with intermediate quality, and 0 – 3 stars showed low quality. Click here for additional data file. Copyright: © 2018 Afriansyah A et al. 2018 Data associated with the article are available under the terms of the Creative Commons Zero "No rights reserved" data waiver (CC0 1.0 Public domain dedication).

## Discussion

Nowadays, clinicians have use PSA not only for screening for PCa, but also for follow up of patients after the treatment. The PSA indicates PCa condition following radical treatment in localized disease, and hormonal treatment in metastatic condition. PSA has a prognostic value, and now has been widen to several parameters such as PSA nadir, TTN, PSA doubling time, and PSA response after the treatment. There is controversy among previous study about the utilization of the PSA kinetic after hormonal treatment for predicting the progression to CRPC and survival.

The meta-analysis performed in this study did not find an association between survival and high initial PSA. Significant heterogeneity was observed due to scattered cut off points of high initial PSA amongst the studies included. Several studies found significant association of initial PSA and survival in univariate analysis, but lost significant after multivariate analysis. This condition showed us the aggressiveness of the cancer has not reflected by PSA alone, and other measures such as Gleason score, PSA nadir, and PSA decline may need to be considered ^10, 15^. This finding was different in localized diseases. High initial PSA reflects disease burden and was found to be correlated with the pathological stage, Gleason score, and the risk of metastasis. The National Comprehensive Cancer Network (NCCN) guideline stratified the risk of localized disease based on PSA and that influences the treatment choice ^27^.

The significant findings of this study showed that lower PSA nadir was associated with good prognosis after ADT treatment. However, due to the variety of PSA nadir threshold, we could not conclude the best optimal threshold of PSA nadir. Most of the papers in this meta-analysis were using below 0.2 ng/ml PSA nadir. Morote *et al.* analyzed 185 patients with metastatic prostate cancer and they found nadir PSA above 0.2 ng/ml was associated with 20 times likelihood progression to CRPC ^28^. Moreover, Stewart *et al.* analyzed patient who received ADT due to biochemical recurrence after radical prostatectomy or radiation therapy, and they suggested PSA nadir above 0.2 ng/ml was associated with significant progression and mortality ^29^. Keizman *et al.* used a different cut off for PSA nadir. They were using below 0.1 ng/ml because they found 4 times increased likelihood of biochemical or clinical progression in patients treated with intermittent ADT due to relapse after radical treatment ^30^.

Our findings found an association between longer time to get nadir PSA and survival. Longer time to nadir was associated with good prognosis. A study by Chung *et al.* found longer time to achieve nadir was a good prognosis for postoperative or post-radiation failure patients receiving ADT ^31^. Possible mechanisms of longer time to nadir associated with a good prognosis was associated with differentiation of PCa cells. Rapid reduction of hormone sensitive cancer cells may induce an environment for the development of hormone resistant PCa cells. In addition, PCa cells that have potential to differentiate into castration resistant cell show a rapid reduction of PSA due to ablation of the androgen receptor. Thus, rapid reduction of PSA is associated to development of CRPC and has a poor prognosis ^32^. This phenomenon is opposite to organ confined PCa receiving radical prostatectomy. In this setting, rapid decline of PSA result is associated with a better prognosis ^33^.

This study has some methodological limitations. We did not analyze the method of administration of ADT due to heterogeneity of ADT administration and that might be influenced survival. Some of the PSA kinetics evaluated in this meta-analysis had significant high heterogeneity. The strengths of this study include (i) a high quality of study based on NOS scale; (ii) meta-analysis just included study with multivariate analysis (iii) several parameters that were associated with the survival were found in this study and might be evaluated in the future research.

## Conclusion

In this study, the intial PSA before administering ADT did not influence the PFS or OS/CSS. Higher PSA nadir during ADT treatment was associated with shortened progression time and survival. A longer time to nadir is a good prognosis of progression and survival of mPCA treated with ADT.

## Data availability

The data referenced by this article are under copyright with the following copyright statement: Copyright: © 2018 Afriansyah A et al.

Data associated with the article are available under the terms of the Creative Commons Zero "No rights reserved" data waiver (CC0 1.0 Public domain dedication). 

**Dataset 1.** Quality assessment (based on NOS), hazard ratio, and standard error of studies included in initial PSA parameter. For quality assessment a maximum 7 stars could be scored; 6 or 7 stars considered as high quality study, 4 – 5 stars corresponded with intermediate quality, and 0 – 3 stars showed low quality. 10.5256/f1000research.14026.d195553 ^34^

**Dataset 2**. Quality assessment (based on NOS), hazard ratio, and standard error of studies included in PSA nadir parameter. For quality assessment a maximum 7 stars could be scored; 6 or 7 stars considered as high quality study, 4 – 5 stars corresponded with intermediate quality, and 0 – 3 stars showed low quality. 10.5256/f1000research.14026.d195573 ^35^

**Dataset 3**. Quality assessment (based on NOS), hazard ratio, and standard error of studies included in time to nadir parameter. For quality assessment a maximum 7 stars could be scored; 6 or 7 stars considered as high quality study, 4 – 5 stars corresponded with intermediate quality, and 0 – 3 stars showed low quality. 10.5256/f1000research.14026.d195577 ^36^

## Supplementary material

Supplementary File 1: Completed PRISMA checklist.
