# Supplementary material for: Prostate specific antigen (PSA) kinetic as a prognostic factor in metastatic prostate cancer receiving androgen deprivation therapy: systematic review and meta-analysis
Source: F1000Res. 2018 Feb 28;7:246. [Version 1] doi: 10.12688/f1000research.14026.1 (PMC5964636; doi:10.12688/f1000research.14026.1)
Supplement: Peer review history

# Peer review: Referee response for version 1

**DOI:** 10.5256/f1000research.15244.r33134

**Published:** 2018-05-21

**Authors:** Bannakij Lojanapiwat

**Affiliations:** 1 Department of Surgery, Chiang Mai University, Chiang Mai, Thailand

This article demonstrated the importance of PSA kinetic in prognosis of prostate cancer that received ADT.

 1. One of important PSA kinetic is PSA doubling time; this should be in the analysis if possible.

 2. The sentence in discussion session” This condition showed us the aggressiveness of the cancer has not reflected by PSA alone, and other measures such as Gleason score, PSA nadir, and PSA decline may need to be considered.” As we know that Gleason score as the aggressiveness of cancer is one of very important factors in all CSS and OS, this should be more detail written in text.

 3. Volume of tumor is very important in combination upfront of chemotherapy /Noval hormonal therapy, this should be added in text that may be related with high initial PSA level.

I have read this submission. I believe that I have an appropriate level of expertise to confirm that it is of an acceptable scientific standard.

# Peer review: Referee response for version 1

**DOI:** 10.5256/f1000research.15244.r33670

**Published:** 2018-05-21

**Authors:** Marniza Saad

**Affiliations:** 1 Clinical Oncology Unit, Faculty of Medicine, University of Malaya, Kuala Lumpur, Malaysia

This article seeks to show correlation between PSA kinetics (initial PSA, PSA nadir, time to nadir) and outcome in patients with metastatic prostate cancer (mPCa) receiving initial treatment with androgen-deprivation therapy (ADT) in terms of survival (PFS, CSS, OS). Seventeen studies were included based on the inclusion and exclusion criteria. The results showed that survival was correlated with PSA nadir and time to nadir (TTN) but not with initial PSA level. Low PSA nadir and long TTN are associated with improved survival. However, due to variation across the studies, no conclusion can be made on the cut-off level of PSA nadir and TTN.

 Overall it was well written. But some areas require review / modification:

1. Methods
  - The inclusion criteria (ii) states that patients were treated with ADT (orchiectomy / LHRH agonists with or without anti-androgen). However, based on Table 1 there are two studies that used anti-androgen monotherapy ^1^^-^^2^. Anti-androgen monotherapy is not included in the criteria.
  - The studies seem to be rather heterogeneous in terms of the outcome measured and the definition of PSA kinetics. Table 1 summarized the studies nicely. But there are inconsistencies in the information given on individual studies in survival outcome column. I guess these are the outcome as reported by the studies. But for the purpose of this review, it would be useful to include on how the parameters studied in this review were defined / analysed in the individual studies i.e. the measurement of PSA kinetics and their definitions / cut-off. 
2. Results
  - There is variation in the survival outcome parameter reported by the studies - some reported median survival while others reported survival at various time-points e.g. 5-year OS. 
  - CSS has been combined with OS (Figures 2,3 & 4). I think this is not ideal since they do not refer to the same outcome but I assume this was done because the number of studies would be too small if they were to be assessed and reported separately.
  - Need to check on the validity of the above with statistician.
3. Discussion
  - In para 4, it was stated that 'Our findings found an association between longer time to get nadir PSA and survival’. I suggest some comments are made on the reasonable time-points to categorise between short and long TTN to guide readers.
  - Since there are quite a variation across the studies in the types of ADT used (ADT alone, ADT + anti-androgen, anti-androgen alone), good to add some comments on this and what data we may have on possible impact on PSA kinetics and survival.
  - In the current era where chemohormonal and ADT in combination with abiraterone acetate have become the new standard of care for metastatic castrate sensitive prostate cancer, good to add some comments on this as we may not know how much the PSA kinetics may be prognostic of survival outcome in patients who are treated with these approaches instead of ADT alone and how the outcome of this review may or may not be applicable to these patients. 
4. There are some errors in grammar / sentence structure / choice of words throughout the manuscript that should be reviewed and corrected. Some examples:
  - Page 3 Introduction para 2: “In clinical practice……. PSA kinetics….might became a predictor of survival in several retrospective and clinical trial studies.”, should be written as “In clinical practice……. PSA kinetics….have been shown to be predictors of survival in several retrospective and prospective clinical studies.”
  - Page 3 Methods under Search Strategy section: “Two researchers…were indecently assessing the title and abstract of the paper. They agreed the studies included in the meta-analysis.” This could perhaps be written as “The titles and abstracts of the papers were extensively assessed by two researchers. The studies for meta-analysis were selected based on the inclusion and exclusion criteria.”.
  - Page 9 Discussion para 2: “Several studies found….., but lost significant after multivariate analysis.”, can be written as “Several studies found….., but the association was not significant after multivariate analysis.” or “Several studies found….., but lost its significance after multivariate analysis.”,
5. Some figures were shown on page 7 - Datasets 1-3, but they are not explained / referred to in the text. These seem to be redundant. They are already documented on page 9 after the Conclusion.

I have read this submission. I believe that I have an appropriate level of expertise to confirm that it is of an acceptable scientific standard, however I have significant reservations, as outlined above.

## References

[1] NayyarRSharmaNGuptaNP: Prognostic factors affecting progression and survival in metastatic prostate cancer. .2010;84(2) : 10.1159/000277592 159-63 10.1159/000277592 20215819

[2] TomiokaATanakaNYoshikawaMMiyakeMAnaiSChiharaYOkajimaEHirayamaAHiraoYFujimotoK: Nadir PSA level and time to nadir PSA are prognostic factors in patients with metastatic prostate cancer. .2014;14: 10.1186/1471-2490-14-33 33 10.1186/1471-2490-14-33 24773608PMC4018264

# Peer review: Referee response for version 1

**DOI:** 10.5256/f1000research.15244.r34017

**Published:** 2018-05-15

**Authors:** Hong-Jun Li

**Affiliations:** 1 Department of Urology, Peking Union Medical College Hospital, Peking Union Medical College, Chinese Academy of Medical Sciences, Beijing, China

In this meta-analysis, the authors analyzed the prognostic value of PSA kinetics in patients with mPCa. They concluded that higher PSA nadir during ADT treatment was associated with shortened progression time and survival. Although the topic was not new, the review has archival value. I have several concerns about the MS and I suggest major revision. 

1. In the aim of the abstract, “hormonal therapy” should be replaced by “ADT” with regard to the homogeneity of the terms.
2. In the introduction part, an explicit statement of the participants, interventions, comparisons, outcomes, and study design (PICOS) is essential.
3. The ADT methods and cut-off point about the PSA indicators varied from different studies, which can result in potential bias. This limitation should be focused and comprehensively discussed.
4. There were several grammar mistakes in the MS. For example, In the table 1. “Significance Prognostic Factor” should be “Significant Prognostic Factor”.

I have read this submission. I believe that I have an appropriate level of expertise to confirm that it is of an acceptable scientific standard, however I have significant reservations, as outlined above.
